# *Mycobacterium porcinum* Skin and Soft Tissue Infections After Vaccinations — Indiana, Kentucky, and Ohio, September 2018–February 2019

**DOI:** 10.15585/mmwr.mm7042a3

**Published:** 2021-10-22

**Authors:** Erin F. Blau, Andrea Flinchum, Kathryn L. Gaub, Kathleen P. Hartnett, Michael Curran, Virginia K. Allen, Allison Napier, Elisabeth M. Hesse, Anne M. Hause, Rachel Cathey, Christine Feaster, Marika Mohr, Sietske de Fijter, Sarah Mitchell, Heather A. Moulton-Meissner, Isaac Benowitz, Kevin B. Spicer, Douglas A. Thoroughman

**Affiliations:** ^1^Epidemic Intelligence Service, CDC; ^2^Department for Public Health, Kentucky Cabinet for Health and Family Services; ^3^Indiana State Department of Health; ^4^Division of Healthcare Quality Promotion, National Center for Emerging Zoonotic and Infectious Diseases, CDC; ^5^Montgomery County Health Department, Mount Sterling, Kentucky; ^6^Ohio Department of Health; ^7^Career Epidemiology Field Officer Program, CDC.

During December 2018–February 2019, a multistate investigation identified 101 patients with vaccination-associated adverse events among an estimated 940 persons in Kentucky, Indiana, and Ohio who had received influenza; hepatitis A; pneumococcal; or tetanus toxoid, reduced diphtheria toxoid, and acellular pertussis (Tdap) vaccines at the workplace during September 11–November 28, 2018. These vaccines had been administered by staff members of a third-party health care company contracted by 24 businesses. Company A provided multiple vaccine types during workplace vaccination events across 54 locations in these adjoining states. Injection-site wound isolates from patients yielded *Mycobacterium porcinum*, a nontuberculous mycobacteria (NTM) species in the *Mycobacterium fortuitum* group; subtyping using pulsed-field gel electrophoresis of all 28 available isolates identified two closely related clusters. Site visits to company A and interviews with staff members identified inadequate hand hygiene, improper vaccine storage and handling, lack of appropriate medical record documentation, and lack of reporting to the Vaccine Adverse Event Reporting System (VAERS). Vaccination-associated adverse events can be prevented by training health care workers responsible for handling or administering vaccines in safe vaccine handling, administration, and storage practices, timely reporting of any suspected vaccination-associated adverse events to VAERS, and notifying public health authorities of any adverse event clusters.

On December 4, 2018, a local health department notified the Kentucky Department for Public Health (KDPH) of three patients who had been evaluated at a local public health clinic for injection-site skin abscesses that occurred after receipt of workplace vaccinations administered by company A. The local health department contacted company A regarding these events and determined that company A had received similar reports from additional patients in early November 2018, but had not reported these events to VAERS or local public health authorities. The local health department instructed company A to immediately cease administration of all vaccines, file VAERS reports, and sequester all remaining vaccines and supplies. 

On December 6, 2018, KDPH issued a health alert notice to notify local health care providers of vaccination-associated adverse events that occurred in the five counties where company A reported conducting vaccination clinics at seven businesses after September 1, 2018. Health care providers were also provided with recommendations for medical evaluation, and were requested to report any adverse events to KDPH. The health alert notice was reissued statewide on December 13, 2018.*

An investigation was subsequently initiated by KDPH to identify cases, establish cause, and prevent further infections. During December 2018, KDPH investigators conducted two site visits to company A. Interviews conducted with company A’s owner and staff members elicited information about vaccine storage, handling, and administration practices as well as protocols regarding hand hygiene and infection control. Investigators obtained vaccination clinic and patient records and collected predrawn syringes with doses of influenza and hepatitis A vaccines and open vaccine multidose vials.^†^ Investigators also collected tap water samples and swabs of surfaces where vaccines were stored, drawn up into syringes, and packed into coolers. All samples were sent to CDC for culture and vaccine antigen detection. Vaccine manufacturer and lot numbers were collected and reported to CDC for review of VAERS reports from other providers.

Details of company A vaccine administration were documented for 355 persons from workplace vaccination clinics at the seven identified businesses. No adverse events associated with the vaccine manufacturers or lot numbers had been reported to VAERS. From observations during company A site visits and interviews with the company owner and staff members, investigators identified breaches in hand hygiene protocols and deviations from recommended vaccine storage and administration practices (*1*). Company A’s owner did not report use of a diluent during vaccine preparation. During vaccination events, hand sanitizer was not used, nor were hands routinely washed, even at events with sink access. Vaccines were stored without temperature monitoring in the office and during off-site vaccination events. Vaccines were predrawn from multidose vials into individual syringes at company A; predrawn syringes were stored for hours to weeks before vaccines were administered to patients. Unlabeled syringes were stored in plastic bags with vaccine type and lot numbers written on the bag. Multidose vaccine vials were stored with food in a compact, dormitory-style refrigerator not recommended for vaccine storage (*1*). The owner and employees lacked clinical licensure and had no formal training in vaccine storage, handling, or administration. Although company A staff members were operating under a physician’s license, no evidence of direct physician oversight was available.

KDPH investigators notified the seven businesses first identified by company A of the ongoing outbreak investigation and interviewed a representative from each business to confirm and supplement information provided by company A, including vaccination dates, number of persons who received vaccines, and number of persons reporting postvaccination symptoms. From these surveys, investigators learned of 17 additional businesses that company A had failed to report to investigators, including facilities in Indiana and Ohio. KDPH notified the Indiana State Department of Health and the Ohio Department of Health, and a multistate investigation was initiated. This activity was reviewed by CDC and was conducted consistent with applicable federal law and CDC policy.^§^

The investigation identified 24 businesses, including the initial seven, that had contracted company A to provide vaccinations at 54 locations across Indiana, Kentucky, and Ohio. Among an estimated 940 persons who received workplace vaccinations during September 11–November 28, 2018, vaccination-associated adverse events occurred in 101 persons. The respective state health departments sent letters to all businesses for distribution to vaccine recipients, notifying them of the risk for vaccination-associated adverse events, advising them to seek medical care for signs or symptoms, and to request that persons report adverse events to their state health department to receive additional guidance regarding medical treatment and revaccination.

Persons reporting vaccination-associated adverse events to their state health department were interviewed and asked about vaccine administration sites, dates, type of vaccines received, symptoms, and any medical treatment received. A case was defined as a self-reported vaccination-associated adverse event characterized by severe redness or swelling, nodule, pustule, abscess, or drainage at the injection site in a vaccine recipient within 150 days of vaccination by company A after September 1, 2018. 

Overall, 179 persons contacted their state health department and completed interviews; among these persons, 101 (56.4%) had a vaccination-associated adverse event that met the case definition, with a median symptom onset of 14 days (range = 0–126 days) after injection (Table). Persons with vaccination-associated adverse events were vaccinated during September 27–November 28, 2018 (with majority of persons vaccinated on either October 3 or October 8); symptom onset dates ranged from October 3, 2018, to February 6, 2019 (Figure 1). Frequently reported symptoms were nodule (97; 96.0%), redness (91; 90.1%), and pain (85; 84.2%) at the injection site. Seventy-seven persons (76.2%) sought medical care for their symptoms, and 35 (34.6.%) reported incision and drainage procedures. Clinical specimens collected by providers were sent to public health laboratories for culture; 30 specimens yielded *M. porcinum *and 28 available specimens were sent to CDC’s environmental and applied microbiology laboratory. Pulsed-field gel electrophoresis of *M. porcinum* isolates yielded two closely related clusters with one band difference; isolates within each cluster are indistinguishable (Figure 2).

**TABLE:** Demographic and clinical characteristics of persons reporting vaccination-associated adverse events* after receipt of vaccine by company A^†^ — Indiana, Kentucky, and Ohio, September 2018–February 2019

Characteristic	No. (%)	
	Overall (N = 101)^§^	*Mycobacterium fortuitum*-group culture (n = 26)^¶^
**Age, yrs, mean (range)**	49 (24–79)	46 (24–65)
Male sex	49 (48.5)	10 (38.5)
**State of residence**		
Indiana	4 (4.0)	0 (—)
Kentucky	71 (70.3)	25 (96.2)
Ohio	26 (25.7)	1 (3.8)
Incubation period,** days, median (range)	14 (0–126)	21 (0–79)
**No. of vaccines received, median (range)**	1 (1–4)	2 (1–4)
**Vaccines received^††^**		
Influenza	91 (90.1)	22 (84.6)
Hepatitis A	54 (53.5)	17 (65.4)
Pneumococcal	12 (11.9)	5 (19.2)
Tdap	3 (3.0)	2 (7.7)
**Vaccine administration site**		
Right arm	27 (26.7)	7 (26.9)
Left arm	27 (26.7)	4 (15.4)
Both arms	47 (46.5)	15 (57.7)
**Reaction site** ^§§^		
Right arm	41 (40.6)	9 (34.6)
Left arm	39 (38.6)	7 (26.9)
Both arms	21 (20.8)	10 (38.5)
**Reported signs and symptoms**		
Nodule	97 (96.0)	25 (96.2)
Redness	91 (90.1)	25 (96.2)
Pain	85 (84.2)	25 (96.2)
Drainage	58 (57.4)	15 (57.7)
Lymphadenitis	8 (7.9)	3 (11.5)
Fever	8 (7.9)	1 (3.8)
Chills	4 (4.0)	0 (—)
Lymphangitis	4 (4.0)	0 (—)
**Reported medical treatment**		
Sought medical care	77 (76.2)	24 (92.3)^¶¶^
Incision and drainage by a medical professional	35 (34.7)	17 (65.4)
Surgical excision by a medical professional	13 (12.9)	7 (26.9)

**Abbreviation:** Tdap = tetanus toxoid, reduced diphtheria toxoid, and acellular pertussis vaccine.

* A case was defined as a vaccination-associated adverse event characterized by severe redness or swelling, nodule, pustule, abscess, or drainage at the injection site in a vaccine recipient ≤150 days after vaccination by company A after September 1, 2018.

^†^ A third-party company located in Kentucky had administered multiple vaccine types in workplace vaccination events across Indiana, Kentucky, and Ohio during September 2018–February 2019.

^§^ Denominator for “Overall” column is 101 unless otherwise noted.

^¶^ Denominator represents number of patients with cultures yielding *Mycobacterium porcinum* and having completed an interview.

** Incubation period was calculated using date of first vaccine administration from company A to date of first symptom onset.

^††^ Total vaccines administered is greater than 101 because some persons reported receiving multiple vaccines from company A.

^§§^ A total of 122 adverse events were reported by 101 patients; this does not include recurrent infections.

^¶¶^ At the time of interview, two of the 26 patients with cultures ultimately yielding *M. porcinum* had not yet sought medical treatment (samples were collected at the time of treatment). After interviews and additional guidance, both persons with symptoms sought medical treatment and had their infection sites cultured by a medical provider.

**FIGURE 1:**
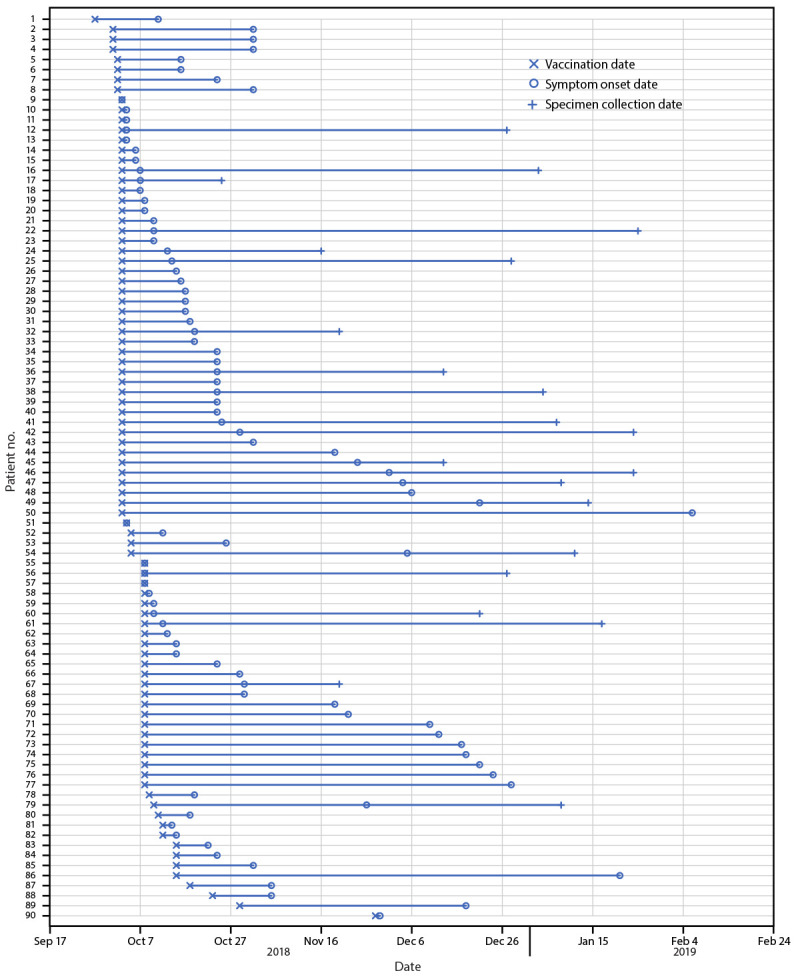
Dates of vaccination, symptom onset, and specimen collection in 90 patients* with vaccination-associated adverse events^†^ after vaccination^§^ by company A — Indiana, Kentucky, and Ohio, September 2018–February 2019 **Abbreviation:** Tdap = tetanus toxoid, reduced diphtheria toxoid, and acellular pertussis vaccine. * Of the 101 interviewed patients, 90 reported both vaccination date and symptom onset date. Of these 90 patients, 21 had cultures that yielded *Mycobacterium porcinum* with specimen collection dates reported. ^†^ A case was defined as a vaccination-associated adverse event characterized by severe redness or swelling, nodule, pustule, abscess, or drainage at the injection site in a vaccine recipient within150 days after vaccination by company A, after September 1, 2018. ^§^ Vaccines administered by company A included influenza, hepatitis A, pneumococcal, and Tdap vaccines.

**FIGURE 2:**
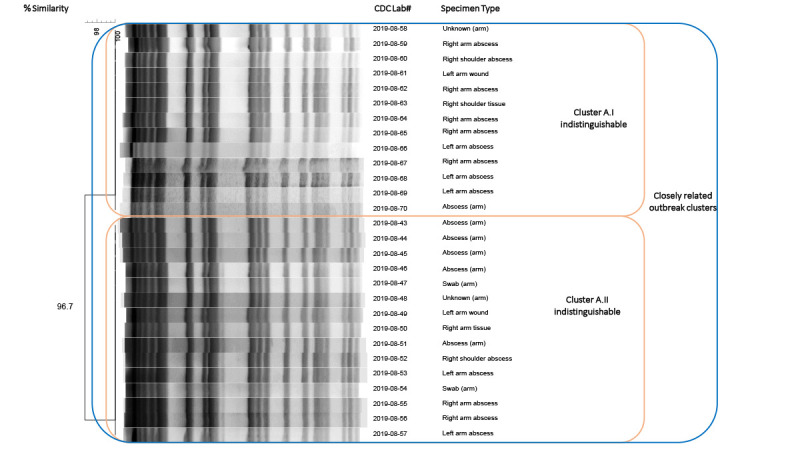
Pulsed-field gel electrophoresis dendrogram* of 28 *Mycobacterium porcinum* specimens isolated from patients vaccinated^†^ by company A — Kentucky and Ohio, September 2018–February 2019 **Abbreviations:** PFGE = pulsed-field gel electrophoresis; Tdap = tetanus toxoid, reduced diphtheria toxoid, and acellular pertussis vaccine. * PFGE patterns of the 28 *Mycobacterium porcinum* clinical isolates, 27 from Kentucky and one from Ohio, showed two closely related clusters with one band difference; isolates within each cluster are indistinguishable. *M. porcinum* is a nontuberculous mycobacteria species in the *Mycobacterium fortuitum* group. ^†^ Vaccines administered by company A included influenza, hepatitis A, pneumococcal, and Tdap vaccines.

Samples collected during company A site visits yielded *Neisseria mucosa* and *Pantoea* sp. from a predrawn syringe of influenza vaccine, and *Streptococcus mitis*, *Rothia mucilaginosa*, and *Staphylococcus hominis* from a predrawn syringe of hepatitis A vaccine. Environmental samples yielded no NTM. Four of six predrawn influenza vaccine syringes had lower than expected hemagglutinin antigen for all four influenza vaccine antigen subtypes by mass spectrometry (Supplementary Table, https://stacks.cdc.gov/view/cdc/110592), and two had no detectable hemagglutinin antigen.

On January 10, 2019, KDPH notified the Kentucky Board of Medical Licensure of the investigation involving a Kentucky licensed physician (the sole ordering and supervising physician of company A). The investigation focus was delegation of vaccination responsibilities to unlicensed personnel with insufficient supervision and training, improper handling of vaccines, and inadequate medical record keeping. KDPH, the Ohio Department of Health, and the Indiana State Department of Health alerted health care providers and provided recommendations for evaluation and care of affected persons. On February 1, 2019, KDPH issued a press release to reach additional persons who received vaccinations from company A. It warned of potential delayed injection-site infections, advised persons experiencing vaccination-associated adverse events to seek medical care, and recommended revaccination. Education concerning proper vaccine storage and handling for health care workers in Indiana, Kentucky, and Ohio is ongoing.

## Discussion

Improper storage, handling, and administration of vaccines were linked to an outbreak of skin and soft tissue infections with *M. porcinum* bacteria among persons who received workplace vaccinations from unlicensed staff members of a third-party health care company that was contracted by businesses in three states. The investigation included tracking vaccine manufacturers and lot numbers of the different vaccines stored at company A and administered during workplace vaccination events. Findings from the epidemiologic investigation and molecular typing of samples from predrawn syringes indicated a common source, suggesting that contamination occurred during syringe preparation. Contamination during syringe preparation was likely worsened by inappropriate storage (days to weeks) in predrawn syringes and at temperatures outside of manufacturer guidance. This finding was further supported by the absence of VAERS reports by other providers associated with these manufacturers or lot numbers. Furthermore, low vaccine antigen levels detected in predrawn syringes of influenza and hepatitis A vaccines suggest that administered vaccines might have been impotent and ineffective. Low or undetectable antigen levels in vaccine samples support the theory of a single diluent that might have been introduced during preparation, thereby reducing vaccine antigen levels found in tested predrawn syringes, though none of the four involved vaccines require reconstitution or dilution and company A reported use of a diluent. Low or undetectable antigen levels also support the theory of a contaminant common to all vaccines and might also be the result of vaccine degradation from storage at incorrect temperatures.

This investigation prompted evaluations of vaccine administration training practices and policies in each of the three states. These evaluations placed particular emphasis on assessing the delegation by medical providers of vaccination administration to lay staff members. Vaccine storage and handling errors can result in decreased vaccine potency and reduced effectiveness, limiting immune response and reducing community protection from vaccine-preventable diseases (*1*). Inactivated vaccines require refrigerator storage temperatures of 35°F–46°F (2°C–8°C) to maintain potency. All vaccine storage units must have a temperature monitoring device (e.g., digital data logger), which provides accurate temperature information and details of any temperature excursions outside the recommended storage range (*2*). The compact refrigerators that were used by company A provide inconsistent temperatures and are not recommended for vaccine storage (*1*). CDC guidance specified that vaccines should only be drawn at the time of administration or after arriving at a mass vaccination event, not predrawn and stored in general-use syringes, and remaining vaccines in predrawn syringes should be discarded at the end of each day (*2*).

NTM are opportunistic pathogens naturally found in environmental sources, including soil, dust, drinking water, and water and ice from refrigerators (*3*). Some states adopted reporting of extrapulmonary NTM cases in 2017 (*4*); however, individual extrapulmonary NTM cases are not reportable conditions in Indiana, Kentucky, or Ohio. Vaccination-associated adverse events reporting delays were caused by both lack of regulations requiring reporting of individual extrapulmonary NTM infections and failure to submit timely reports of adverse events to VAERS by company A. In addition, incomplete record keeping by company A and incomplete reporting of businesses where company A conducted clinics, likely resulted in cases being missed. Earlier detection would have assisted investigators in identifying cases, businesses, and transmission source. Jurisdictions that have added NTM to regulations for reportable diseases have improved their ability to detect and respond to health care–associated outbreaks (*5*,*6*).

This rare outbreak of postvaccination injection site NTM infections highlights the vital role of trained staff members in proper vaccine storage, handling, and administration, and in reporting adverse events to public health authorities. This outbreak was entirely preventable; with proper storage, handing, and administration, vaccines are safe and effective. Persons experiencing postvaccination adverse events should seek medical care, and clinicians caring for persons who experience such adverse events should submit reports through VAERS (https://vaers.hhs.gov, 1-800-822-7967) and contact their local and state health departments.

**Summary**

**What is already known about this topic?**

Adherence to vaccine storage, preparation, and administration guidelines is critical to ensure safe, effective vaccination. Improper vaccine handling can increase the risk for adverse events.

**What is added by this report?**

A multistate investigation identified 101 patients with vaccination-associated adverse events, including 30 with confirmed nontuberculous mycobacteria infection (vaccines received included influenza; hepatitis A; pneumococcal; or tetanus toxoid, reduced diphtheria toxoid, and acellular pertussis vaccines). Improper vaccine storage, handling, and administration by inadequately trained personnel contributed to injection-site infections and other adverse events.

**What are the implications for public health practice?**

Correctly trained health care workers play a vital role in proper vaccine storage, handling, and administration. Timely reporting to the Vaccine Adverse Event Reporting System and notifying public health authorities of any adverse event clusters are important to detecting vaccination-associated adverse events.

## Footnotes

*
https://www.lexingtondoctors.org/2018/12/14/vaccination-abscess-provider-alert/

†
https://www.cdc.gov/injectionsafety/providers/provider_faqs_multivials.html

§ 45 C.F.R. part 46, 21 C.F.R. part 56; 42 U.S.C. Sect. 241(d); 5 U.S.C. Sect. 552a; 44 U.S.C. Sect. 3501 et seq.
