# Erratum: Vol. 70, No. 42

**DOI:** 10.15585/mmwr.mm7044a3

**Published:** 2021-11-05

In the report “*Mycobacterium porcinum* Skin and Soft Tissue Infections After Vaccinations — Indiana, Kentucky, and Ohio, September 2018–February 2019,” on page 1474, in the second column, first continued paragraph, the sentence beginning on line 12 should have read, “Low or undetectable antigen levels in vaccine samples support the theory of a single diluent that might have been introduced during preparation, thereby reducing vaccine antigen levels found in tested predrawn syringes, though none of the four involved vaccines require reconstitution or dilution and company A **did not report** use of a diluent.”

